# Rumen microbiota regulates IMF deposition in Xizang sheep by activating the *PPARγ* transcription factor: a rumen-muscle axis perspective

**DOI:** 10.1128/msystems.01557-24

**Published:** 2025-03-28

**Authors:** Cheng Pan, Junru Pan, Yangzong Zhaxi, Haiyan Li, Zhenzhen Zhang, Feng Guan, Jiacuo Jinmei, Zhaxi Baijiu, Sangzhu Baima, Quzhu Yixi, Tianzeng Song, Wangsheng Zhao

**Affiliations:** 1 School of Life Sciences and Agri-forestry, Southwest University of Science and Technology 91609, Mianyang, Sichuan, China; 2 Institute of Animal Science, Xizang Academy of Agricultural and Animal Husbandry Science, Lhasa, Xizang, China; 3 Key Laboratory of Animal Genetics and Breeding on Xizang Plateau, Ministry of Agriculture and Rural Affairs, Lhasa, Xizang, China; 4 College of Life Sciences, China Jiliang University 92270, Hangzhou, Zhejiang, China; 5 Xizang Animal Husbandry Station, Lhasa, Xizang, China; 6 Cultural Service Center of Maqian Township, Nagqu, Xizang, China; 7 The Service Station of Agricultural and Animal Husbandry Technical of Baingoin County, Nagqu, Xizang, China; The University of Maine, Orono, Maine, USA

**Keywords:** Xizang sheep, meat flavor, IMF, rumen-muscle axis, multi-omics

## Abstract

The interaction between microbiota and muscle by the rumen-muscle axis and its impact on sheep meat flavor has received little attention. This study selected Xizang sheep under summer and autumn grazing conditions as models for different rumen bacteria and intramuscular fat (IMF) to attempt to address the current research gap. Specifically, the deposition characteristics of IMF and the expression of lipid metabolism genes in Xizang sheep were determined; 16S rDNA sequencing technology and gas chromatography were used to study the rumen microbiota and its metabolic products, short-chain fatty acids (SCFAs); RNA sequencing was used to identify the transcriptome of the rumen epithelium. Based on the above results, we proposed the hypothesis that the flavor of Xizang sheep meat is regulated by the microbiota-rumen-muscle axis. SCFAs produced in the rumen of Xizang sheep are absorbed by the rumen epithelium under the regulation of the solute carrier family genes (*SLC*). SCFAs can directly reach muscle tissue through the circulatory system and then activate the expression of the peroxisome proliferator-activated receptor Gamma (*PPARγ*) gene through the rumen-muscle axis. The expression of fat synthesis genes carnitine palmitoyltransferase II (*CPT2*), fatty acid synthase (*FAS*), patatin-like phospholipase domain-containing 2 (*PNPLA2*), and stearoyl-CoA desaturase 1 (*SCD1*) is correspondingly upregulated, promoting the deposition of IMF in Xizang sheep and thus affecting its flavor. This study introduces the theory of the microbiota-rumen-muscle axis into the research of the flavor of ruminant animal food, comprehensively elucidating the regulatory mechanism of the flavor of Xizang sheep meat.

**IMPORTANCE:**

Our study employed a multi-omics approach to reveal how the rumen microbiota regulate muscle lipid metabolism in Xizang sheep through the activation of the *PPARγ* transcription factor. Importantly, by developing models of Xizang sheep with varying rumen microbial communities and muscle fatty acid profiles, we established the critical role of the microbiota-rumen-muscle axis in determining the flavor of Xizang sheep meat. This finding suggests that modulating the composition of the microbial community could serve as a strategy to improve the flavor of ruminant-derived food products. These insights provide valuable understanding of the complex interactions between rumen bacteria and mutton flavor, offering new approaches for research in this field.

## INTRODUCTION

In recent years, as people’s demands for quality of life continue to rise, consumers are increasingly pursuing the flavor of animal-derived foods (1, 2). Xizang sheep, as one of the main animal-derived foods in the plateau region, have become a pillar of local animal husbandry (3). Therefore, exploring the regulatory mechanisms of Xizang sheep flavor to provide improvement strategies is of great significance. Previous studies have revealed the key role of fatty acids and amino acids in meat flavor and their complex relationship with volatile compounds and non-volatile taste substances (4). Research data also indicated that the nutritional composition of the diet affected the metabolism of fatty acids in sheep (5, 6). Xizang sheep are mainly pastured, and their main feed source is grass. Previous studies have proven that the type of grass affects the fatty acid composition of fattening goat meat (7). As a result, the nutritional elements of forage will alter important components like fatty acids and amino acids in mutton, which will ultimately impact the meat’s flavor.

The gut-muscle axis has been proposed as a way to illustrate the relationship between the host’s muscles and the gut microbiota, with the functions of gastrointestinal bacteria increasingly being explored (8). The gut-muscle axis is one of the signaling pathways in fat metabolism that is now known to be influenced by gut microbes or their metabolites (9, 10). Yet, there is not enough study on how the rumen-muscle axis affects fat metabolism. Nevertheless, there is evidence that the rumen bacteria may have an impact on the metabolic activities of muscles (11, 12). Different proportions of forages in fermented total mixed ration have been shown in experiments involving lambs to modify the rumen microbiota, suggesting a potential connection between significant alterations in the rumen microbiota and variations in the composition of muscle fatty acids (13). Thereby, it is possible to conduct more studies on the relationship between the rumen-muscle axis and the flavor of meat in ruminant animals. The microbial community consists of protozoa, fungi, bacteria, and archaea. Ninety-five percent of the rumen microbiota is made up of bacteria, which produce a lot of short-chain fatty acids (SCFAs), which are transported by the bloodstream and supply the host 75% of its energy (14, 15). Consequently, rumen bacterial research has drawn a lot of interest. Given the importance of the rumen and its microbiota in the digestive system of ruminant animals, a hypothesis has been proposed that the regulation of intramuscular fat (IMF) deposition in ruminants is mainly achieved through the rumen-muscle axis, thereby regulating the flavor of meat.

This study focuses on Xizang sheep grazing in summer and autumn. The fatty acid content of the longissimus dorsi (LD) muscle, the expression of fat metabolism genes, and the concentration of ruminal SCFAs were measured. High-throughput 16S rRNA sequencing technology was employed to investigate the differences in the composition of ruminal bacterial communities in Xizang sheep across different seasons. RNA sequencing (RNA-Seq) technology was utilized to identify differentially expressed genes (DEGs) in the ruminal tissue of Xizang sheep, and Gene Ontology (GO) and Kyoto Encyclopedia of Genes and Genomes (KEGG) analyses were conducted to annotate the functions of DEGs. Through these methods, the study aims to reveal the link between the rumen-muscle axis and the flavor of Xizang sheep meat. This research not only provides a theoretical reference for the application of the rumen-muscle theory in livestock production but also opens up new ideas for improving the meat flavor of Xizang sheep.

## MATERIALS AND METHODS

### Experimental design and sampling

The experiment was carried out in Maqian Village, Bango County, Xizang, China, between June and November 2023. In both summer and autumn, six healthy Sewa sheep with an average age of 1 year and similar body weight were selected from a group of 100 Xizang sheep. Two groups of sheep were created based on the seasons: the summer group (SUM; *n* = 6) and autumn group (AUT; *n* = 6). All sheep in Bango County, Xizang, were grazed on natural grassland at an elevation of roughly 4,750 m above sea level. Food and water were available to the sheep without restriction during the experiment period. Six sheep were slaughtered at the end of the research in the summer (31 August) and fall (30 November) following a 12 h fast. Rumen fluid and LD muscle were then obtained. After being brought to the laboratory and kept at −80°C for further analysis, they were preserved in liquid nitrogen.

### Determination of SCFAs in the rumen

One gram of the frozen rumen fluid sample at 4°C was measured and then transferred to the proper centrifuge tube in order to determine the SCFAs, such as acetic acid, propionic acid, butyric acid, valeric acid, and isovaleric acid, in the feces. Immediately, 2 mL of 0.1% hydrochloric acid was added and mixed. The mixture was left in an ice bath for 25 min. It was then centrifuged for 15 min at 15,000 rpm while keeping the temperature at 4°C. To determine the SCFAs, the supernatant was extracted using a syringe, filtered through a 0.22 µm filter membrane (Millipore, USA), placed into a sample vial, and then injected into a gas chromatograph (Agilent HP 6890 series, USA).

### RNA extraction, cDNA synthesis, and real-time quantitative PCR

Rumen and muscle samples were treated according to standard protocols, and total RNA was extracted using RNAiso Plus reagent (Takara Biotechnology Ltd., Dalian, China). After that, mRNA was reverse transcribed to cDNA using the Takara PrimeScriptTM RT kit and gDNA Eraser. TB Green Premix Ex TaqTM II (Tli RNaseH Plus) and the CFX96 Real Time PCR Detection System (Biorad, Hercules, CA, USA) were used to extract mRNA via real-time quantitative PCR (qPCR). qPCR was conducted using the Taq II (Tli RNaseH Plus) and CFX96 Real Time PCR Detection System (Biorad, Hercules, CA, USA). The 2^-ΔΔCt^ method was used to evaluate relative gene expression. The primer design for genes can be found in Table S1.

### RNA sequencing and analysis

MetWare Biotech (Wuhan, China) used the Illumina NovaSeq 6000 platform (Illumina, San Diego, CA, USA) to perform RNA sequencing. Using the sheep reference genome (https://ftp.ncbi.nlm.nih.gov/genomes/all/GCF/000/298/735/GCF_000298735.2_Oar_v4.0/) as a point of comparison, raw data were filtered to provide pure data before being sequenced using Hisat2 (16). DEGs between the two groups were examined using DESeq (17), and gene reads were quantified using FeatureCounts (18). By utilizing the Benjamini-Hochberg method to alter the *P*-value, false discovery rates (FDRs) were determined. The criteria |log2Fold Change| ≥ 1 and FDR < 0.05 were used to identify DEGs. The Metware Cloud platform (https://cloud.metware.cn) was used for data analysis, including the creation of heat maps, volcanic maps, and GO and KEGG studies.

### DNA extraction

The CTAB method was used to extract the whole genome DNA from samples of rumen fluid. One percent agarose gels were used to measure the concentration and purity of DNA. DNA was analyzed for purity and concentration after being diluted with sterile water to 1 ng/µL based on the concentration. Detailed steps for amplicon generation and PCR product quantification and qualification are described in the literature (19).

### 16S rDNA amplicon sequencing and analysis

MetWare Biotechnology Co., Ltd. (Wuhan, China) used the Illumina NovaSeq 6000 platform (Illumina, San Diego, CA, USA) to sequence a 16S rDNA amplicon from sheep rumen fluid DNA. To create clean data, the collected raw data were spliced and filtered. Deblur was used to do the denoising, which produced amplicon sequence variants (ASVs). Mothur (version 1.48) annotated the ASV sequences for species, and analyses were conducted on taxonomic data and community compositions at several levels (phylum, class, order, family, genus, and species). In order to determine common and unique ASVs among various samples, as well as to evaluate species richness and evenness in the samples, the alpha diversity metrics—Shannon, Simpson, Chao1, ACE, observed ASV, Goods coverage, and PD entire tree—were examined. Based on the weighted UniFrac distance of ASV abundances, the beta diversity between groups was examined using principal coordinate analysis (PCoA), principal component analysis, non-metric multi-dimensional scaling, and unweighted pair-group method with arithmetic means. The *t*-test and Wilcoxon test were used to examine the variations between the two groups’ microbial makeup and community structure. Furthermore, the association between bacteria and DEGs, as well as between bacteria and amino and fatty acids, was evaluated using Spearman’s correlation coefficient.

### Targeted metabolic fatty acid extraction and analysis

Twelve LD muscle samples, which had been taken out of an ultra-low temperature refrigerator at −80°C, were immediately subjected to metabolite extraction. The LD muscle was ground with a grinder (30 Hz for 1 min) until it was powdered. Fifty milligrams of the ground sample was weighed precisely and then 150 µL of the methanol solution and 200 µL of methyl tert-butyl ether were transferred into a fresh EP tube. After 3 min of vortexing, the supernatant was centrifuged for 5 min at 4°C at 12,000 rpm. After pipetting and drying the supernatant with a nitrogen blower for 200 µL, 300 µL of 15% boron trifluoride methanol solution was added. After 3 min of vortexing, it was baked at 60°C for 30 min. After cooling to room temperature, 200 µL of saturated sodium chloride solution and 500 µL of n-hexane solution were added. To prepare for gas chromatography analysis, 100 µL of the n-hexane layer solution was pipetted after centrifugation for 5 min at 4°C at 12,000 r/min and vortexing for 3 min.

### Targeted metabolic amino acid extraction and analysis

Fifty milligrams of the six summer and autumn LD samples was weighed. Then, 500 µL of a 70% methanol aqueous extract that had been chilled beforehand was added to the sample and vortexed for 3 min. After 10 min of 4°C centrifugation at 12,000 rpm, 300 µL of the supernatant was aspirated into a 1.5 mL centrifuge tube. After 30 min at −20°C, the sample was centrifuged at 12,000 rpm for 10 min at 4°C. The centrifuged supernatant was stored at −20°C after 200 μL of it was run through a protein precipitation plate for analysis. At −20°C, the supernatant was kept. Liquid chromatography-tandem mass spectrometry was used for the final analysis.

### Statistical analysis

SPSS (version 21.0) software was used for statistical analysis. Column charts were plotted using GraphPad Prism (9.0) software. Student’s *t* test was used for comparisons between two groups. *P* < 0.05 was considered statistically significant, and *P* < 0.01 was considered highly significant.

### Co-occurrence network analysis

We built co-occurrence networks by choosing microbiota with significant differences at the genus level, significant DEGs, and amino acids and fatty acids in order to understand the relationship between microbes in the rumen and gene expression, amino acids, and fatty acids in muscle. Python software was used to analyze the correlation network using Spearman’s correlation coefficient (20). Cytoscape version 3.0.1 was used to visualize correlations (|rho| > 0.70).

## RESULTS

### Analysis of Xizang sheep body weight changes

As shown in Table 1, there were no significant differences in the initial body weight, final body weight, and average daily gain between the two groups of Xizang sheep. However, compared to the AUT group, the SUM group showed a trend toward increased final body weight and average daily gain.

**TABLE 1:** Xizang sheep body weight changes

Index	AUT	SUM
Initial weight (kg)	35.18 ± 1.06	32.17 ± 0.88
Final weight (kg)	45.03 ± 1.28	48.00 ± 1.05
Average daily gain (g)	115.37 ± 8.3	175.93 ± 4.34

### Rumen SCFA analysis

In animals, especially ruminants, SCFAs are a major manifestation of energy metabolism and have several regulatory functions (21). We discovered that group SUM had a significantly higher concentration of acetic acid than group AUT based on an analysis of the SCFA concentrations in their rumen fluid (*P* < 0.05). In contrast, propanoic acid, butyric acid, isobutyric acid, valeric acid, and isovaleric acid did not show a significant difference between the two groups (Table S2). Moreover, group SUM’s total SCFA (T-SCFA) concentration was higher than group AUT’s (Table S2). The research mentioned above shows that Xizang sheep could store more energy throughout the summer.

### Analysis of rumen microbiota composition

**Fig 1:**
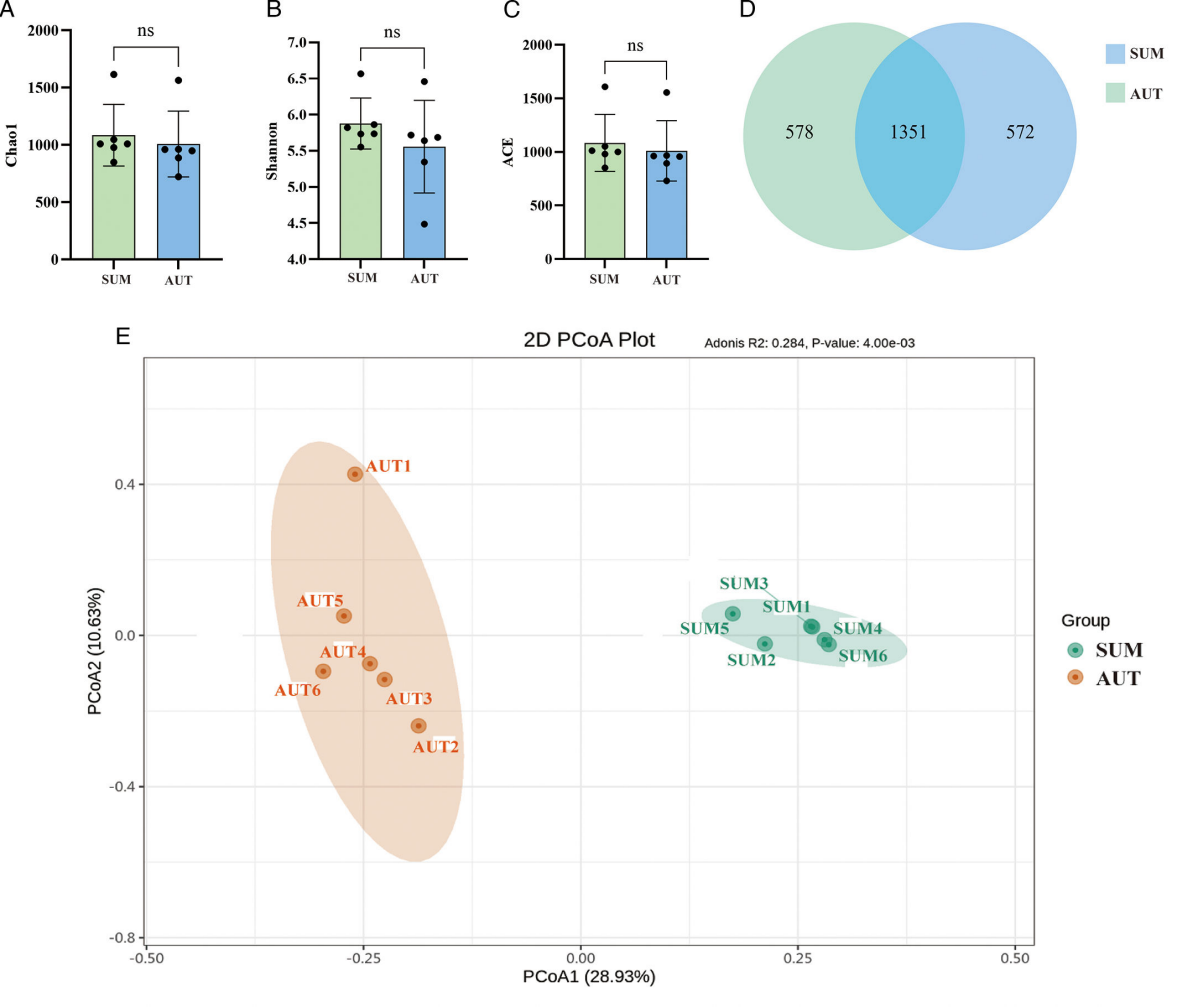
Effects of different seasons on the diversity of rumen microbiota. ns represents no significant difference between groups. (A) Chao1 index. (B) Shannon index. (C) ACE index. (D) Venn diagram. (E) PcoA.

The rumen microbiota diversity and abundance of Xizang sheep were likely similar in the fall and summer, as indicated by the lack of significant variations in the Chao1, Shannon, and ACE indices between the two groups, AUT and SUM (Fig. 1A through C). Between the two groups, a total of 1,351 ASVs were found, with groups SUM and AUT having 572 and 578 identified ASVs, respectively (Fig. 1D). Groups SUM and AUT were found to be separate based on PCoA plots (Fig. 1E).

**Fig 2:**
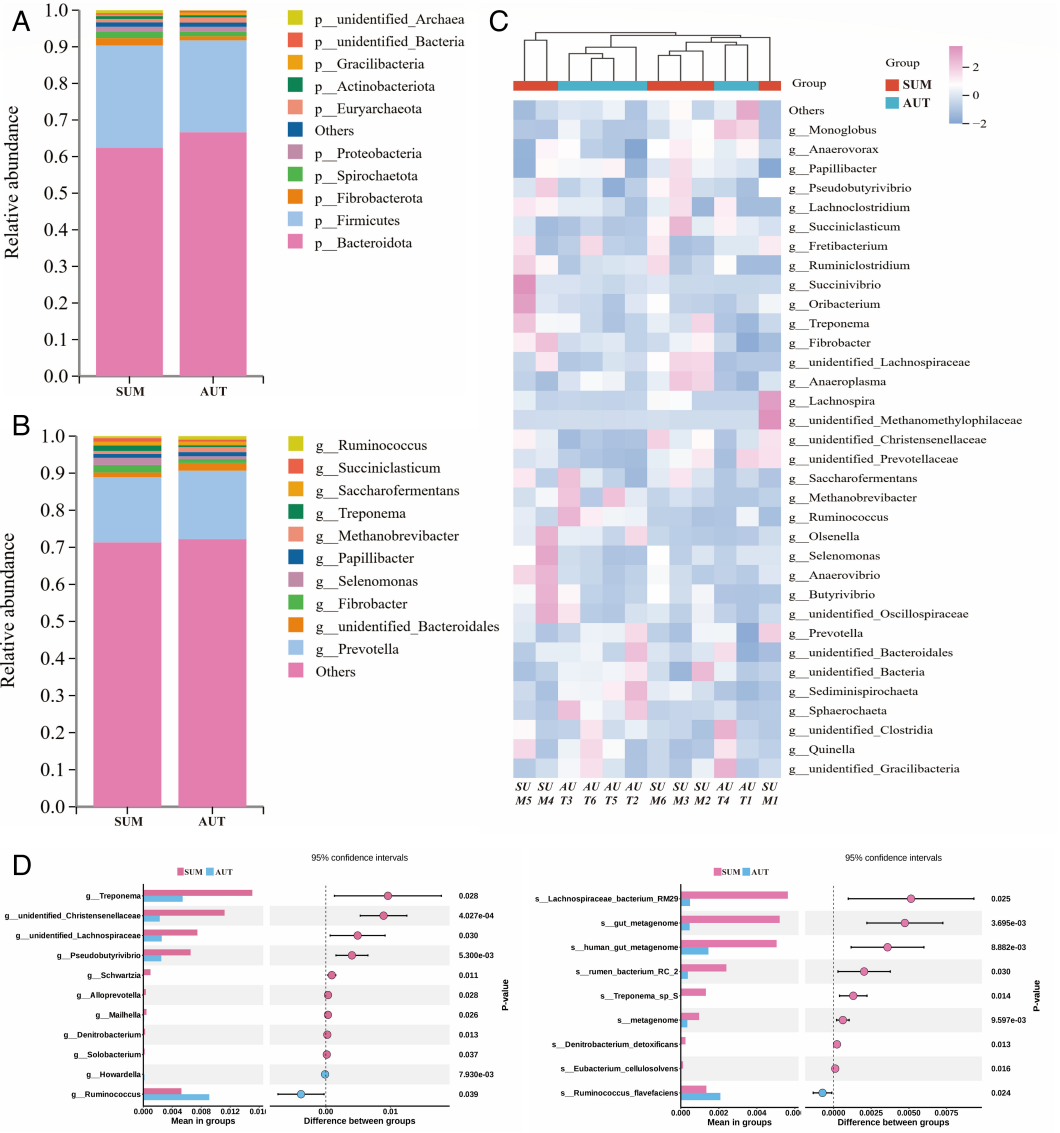
Effects of different seasons on the composition of rumen microbiota. (A) At the phylum level, the composition of the TOP 10 relative abundance of rumen microbes in the two groups. (B) At the genus level, the composition of the TOP 10 relative abundance ratio of rumen microbes in the two groups. (C) Heatmap of rumen microbiota clustering at genus level with relative abundance TOP35. (D) Rumen microbiota with significant differences at genus level and species level.

Firmicutes and Bacteroidota were the most dominant bacteria at the phylum level between the two groups, SUM and AUT, followed by Fibrobacterota, Spirochaetota, Proteobacteria, etc. (Fig. 2A). When comparing group AUT to group SUM, the relative abundance of Bacteroidota increased by 4.28% and that of Firmicutes reduced by 2.89% (Table S3). *Prevotella* and *unidentified Bacteroides* were the most advantageous bacteria at the genus level between the two groups SUM and AUT, followed by *Fibrobacter*, *Selenomonas*, *Papillibacter*, etc. (Fig. 2B). When comparing group AUT to group SUM, the relative abundance of *Prevotella* increased by 0.86% and that of *unidentified Bacteroidales* by 0.81% (Table S4). Furthermore, season had a minimal effect on the rumen microbiota, according to the hierarchical clustering at the microbial genus level of TOP35 (Fig. 2C).

*t*-test between groups AUT and SUM was used to further screen microbes for significant bacteria at the genus and species levels (Fig. 2D). The relative abundance of nine microbes in group SUM was significantly upregulated, including *unidentified_Christensenellaceae*, *Pseudobutyrivibrio*, *Schwartzia*, *Denitrobacterium*, *Mailhella*, *Treponema*, *Alloprevotella*, *unidentified_Lachnospiraceae*, and *Solobacterium* (*P* < 0.05). In group AUT, the relative abundance of two microbes—*Ruminococcus* and *Howardella*—was significantly increased (*P* < 0.05). At the species level, the SUM group exhibited a considerable upregulation of the relative abundance of bacteria like *Lachnospiraceae_bacterium_RM29* and *human_gut_metagenome*, while the AUT group showed a significant upregulation of *Ruminococcus_flavefaciens* (*P* < 0.05).

### DEGs identified in the rumen of AUT and SUM

**Fig 3:**
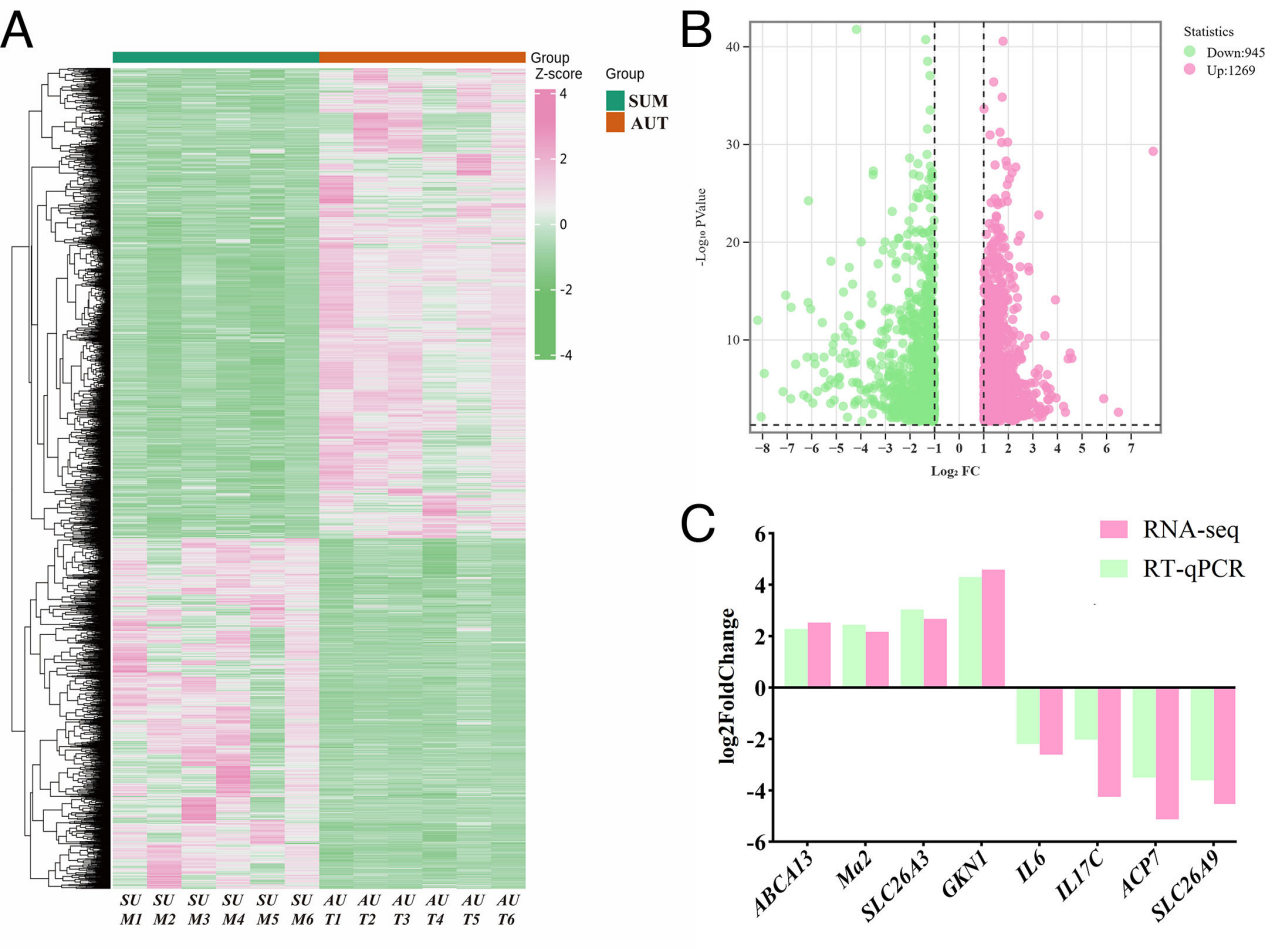
Effects of different seasons on rumen epithelial transcriptome expression profiles and qPCR validation. (A) Heatmap of clustering of two groups with DEGs. (B) Volcano diagram. (C) qPCR was used to verify the mRNA levels of *ABCA13*, *MX2*, *SLC26A3*, *GKN1*, *IL6*, *IL17C*, *ACP7*, and *SLC26A9*.

To evaluate the profiles of gene transcript expression, RNA-seq analysis was employed. Twelve mRNAs from rumen tissue samples were utilized in the cDNA libraries; six came from SUM in the summer and the other six from AUT in the autumn. The findings revealed that there were 2,214 DEGs in total, 1,269 of which were significantly upregulated and 945 of which were considerably downregulated in the rumen epithelial tissues of sheep in groups SUM and AUT (Fig. 3B). Genes with high and low expression levels were found to be clustered together in rumen tissue samples from groups SUM and AUT, respectively, according to further cluster analysis (Fig. 3A). This finding suggests that the season had a unique effect on the expression of rumen gene transcripts. The qPCR findings corroborated the RNA-seq results in terms of the alterations in ATP-binding cassette sub-family A member 13 (*ABCA13*), myxovirus resistance 2 (*MX2*), solute carrier family 26 member 3 (*SLC26A3*), gastrokine 1 (*GKN1*), interleukin 6 (*IL6*), interleukin 17C (*IL17C*), acid phosphatase 7 (*ACP7*), and solute carrier family 26 member 9 (*SLC26A9*) mRNA levels (Fig. 3C).

### The functional analysis of DEGs

**Fig 4:**
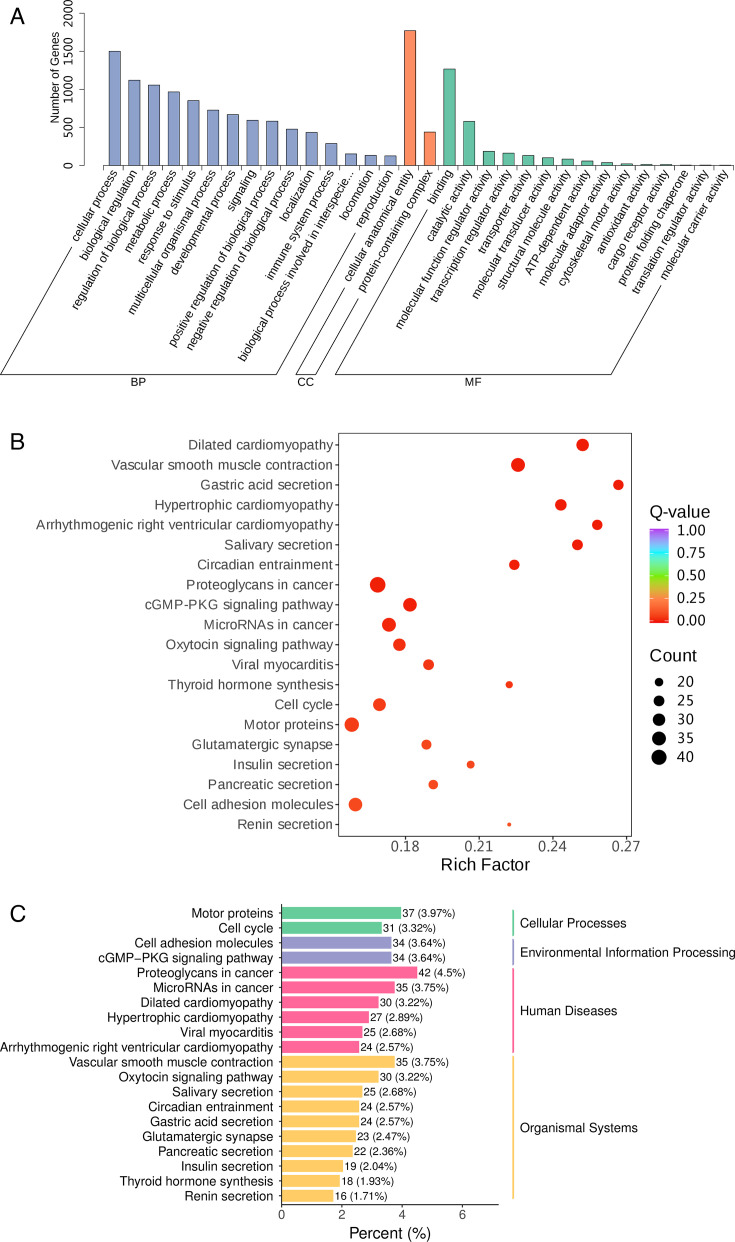
Functional analysis of DEGs in the rumen epithelium. (A) The significantly enriched GO terms were identified for the DEGs, with a threshold set for statistical significance at a *P*-value of less than 0.05. (B) KEGG analysis was conducted for all DEGs. (C) KEGG pathway percentage graph.

DEGs’ functional characteristics were examined using GO and KEGG enrichment analysis. The annotated genes in the GO database were grouped into three categories: molecular function (MF), cellular component (CC), and biological process (BP) (Fig. 4A). Biological regulation (GO:0065007), regulation of biological process (GO:0050789), and cellular process (GO:0009987) are the first three processes that are primarily enriched in the majority of DEGs annotated in BP. Differentially expressed genes annotated in MF are primarily enriched in binding (GO:0005488), molecular function regulator activity (GO:0098772), and transcription regulator activity (GO:0140110); DEGs annotated in CC are primarily enriched in protein-containing complex (GO:0032991) and cellular anatomical entity (GO:0110165) (Fig. 4A). Furthermore, KEGG pathway analysis demonstrated a significant enrichment of TOP5 pathways, which account for 4.5%, 3.97%, 3.75%, 3.75%, and 3.64% of the total and include Proteoglycans in cancer, Motor proteins, MicroRNAs in cancer, Vascular smooth muscle contraction, and Cell adhesion molecules (Fig. 4B and C). Among these, the intake of nutrients by the rumen is indirectly influenced by motor proteins and cell adhesion molecules (22, 23).

### Expression and functional analysis of the *SLC* gene in rumen epithelium

The absorption of nutrients by cells is influenced by the expression of *SLC* genes (24). Ruminants mostly obtain their energy from SCFAs, and a study has indicated that solute carrier family 16 member 1 (*SLC16A1*) is involved in the uptake of SCFAs (25). Thus, we screened for 38 *SLC* genes in 2,214 DEGs found in the rumen epithelial tissues of groups SUM and AUT of sheep. The differentially expressed *SLC* genes in the rumen tissue samples of groups SUM and AUT were found to be clustered together separately, indicating that rumen *SLC* gene expression is specific to the season (Fig. S1A). Of the *SLC* DEGs, 17 were found to be upregulated and 21 to be downregulated by volcano plot analysis (Fig. S1B). When the functional characteristics of DEGs were analyzed using KEGG enrichment analysis, it was shown that the Mineral absorption pathway had the highest concentration of DEGs. This pathway was followed by the Protein digestion and absorption, Bile secretion, Parathyroid hormone synthesis, secretion, and action, and Adipocytokine signaling pathway (Fig. S1C). A complex connection between these genes was shown by correlation analysis of *SLC* genes (Fig. S1D).

### Expression analysis of myosin heavy chain genes and genes related to lipid metabolism

**Fig 5:**
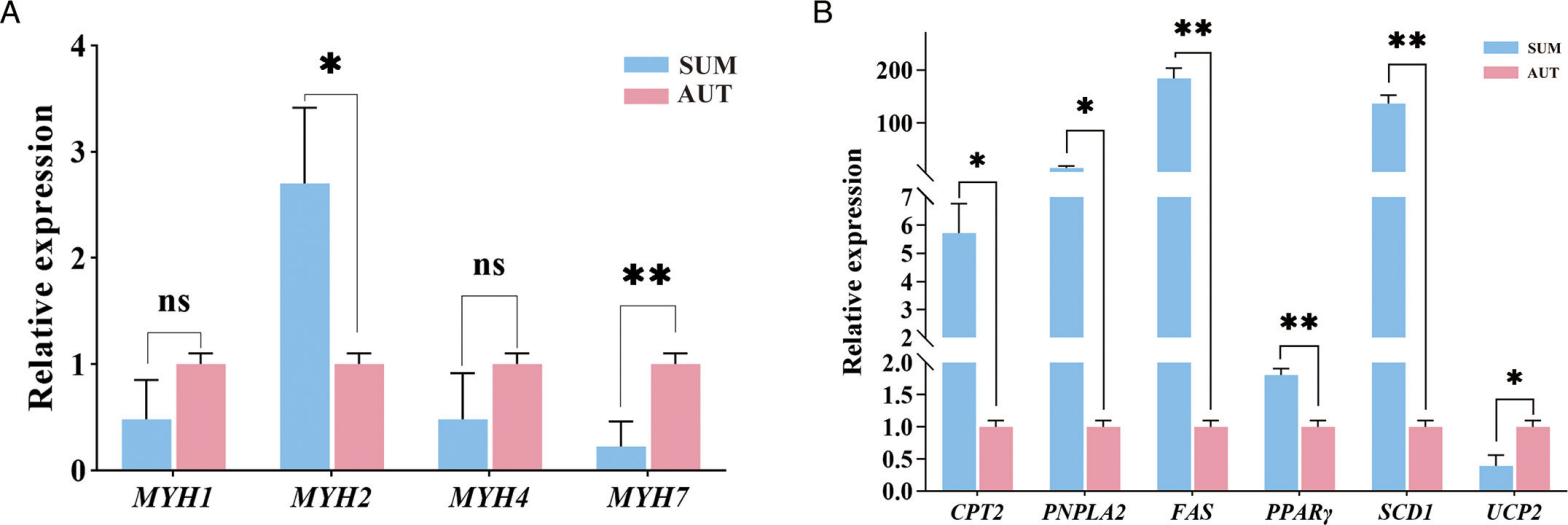
Effects of different seasons on the expression of muscle *MYH* gene and lipid metabolism-related genes. (A) qPCR was used to verify the mRNA levels of myosin heavy chain 1 (*MYH1*), *MYH2*, myosin heavy chain 4 (*MYH4*), and *MYH7*. (B) qPCR was used to verify the mRNA levels of *CPT2*, *PNPLA2*, *FAS*, *PPARγ*, *UCP2,* and *SCD1*.

We used real-time fluorescence quantitative detection of associated genes to examine the variations in muscle quality of the LD of Xizang sheep in various seasons. It was discovered that the myosin heavy chain (*MYH*) gene could control the type of muscle fiber and its properties, which in turn influenced the properties of muscle contraction and the quality of the meat (26). The LD myosin heavy chain 2 gene (*MYH2*) was discovered to be significantly upregulated in group SUM (*P* < 0.05), and group AUT had a significantly upregulated myosin heavy chain 7 gene (*MYH7*) (*P* < 0.01) (Fig. 5A). Furthermore, we explored the expression patterns of genes associated with lipid metabolism. We found that group SUM exhibited significantly higher expression levels of the following genes compared to group AUT: *CPT2* (*P* < 0.05), *PNPLA2* (*P* < 0.05), *FAS* (*P* < 0.01), *PPARγ* (*P* < 0.01), and *SCD1* (*P* < 0.01). In contrast, group AUT had considerably higher levels of *UCP2* gene expression (*P* < 0.05) (Fig. 5B).

### Muscle fatty acid composition

The total fatty acid composition and content in the muscles of Xizang sheep were measured in several seasons in order to confirm the fatty acid content of meat metabolites (Table 2). Group SUM had significantly higher concentrations of C16:1, C18:1n9t, C20:5n3, C22:6n3, and C20:3n6 than group AUT. The consumption of saturated fatty acids (SFAs) has been linked in studies to an increased risk of cardiovascular disease (27); however, there was not a significant difference in SFA concentrations between both groups. Compared to group AUT, group SUM exhibited a notably greater content of monounsaturated fatty acids (MUFA), a fatty acid that is frequently acknowledged in studies as being advantageous for heart health (28). The body needs polyunsaturated fatty acids (PUFAs) for good health, but it cannot produce them on its own; instead, the body must get them through diet (29). The concentrations of PUFAs (n-3 PUFAs and n-6 PUFAs) did not significantly differ between the two groups; however, group SUM showed a trend of rising concentration in comparison to group AUT. These results indicate that the fatty acid profile in the muscles of Xizang sheep during summer is superior to that in autumn.

**TABLE 2:** Effects of different seasons on fatty acid concentrations in the LD (μg/g)^*a*^

Index	AUT	SUM	SEM	*P*-value
C6:0	0.245 a	0.676 b	0.010	0.005
C8:0	0.432	0.938	0.035	0.210
C9:0	0.186	0.399	0.011	0.060
C10:0	1.746	3.468	0.129	0.898
C11:0	0.054	0.107	0.007	0.852
C12:0	0.742	1.662	0.045	0.080
C13:0	0.101	0.206	0.008	0.713
C14:0	10.06	20.44	0.347	0.641
C15:0	2.715	5.483	0.106	0.792
C16:0	240.0	488.8	7.778	0.481
C17:0	9.66	18.40	0.796	0.358
C18:0	181.4	383.1	7.855	0.170
C19:0	0.947	1.878	0.064	0.888
C20:0	1.810	3.738	0.108	0.468
C21:0	0.708	1.409	0.007	0.516
C22:0	0.933	1.894	0.017	0.441
C23:0	0.959	1.918	0.010	0.998
C24:0	1.010	2.039	0.010	0.389
SFA	453.7	936.6	16.32	0.306
C14:1	1.443	2.789	0.108	0.532
C15:1	30.04	57.60	2.093	0.452
C16:1	14.75 a	37.12 b	1.286	0.001
C18:1n9c	171.7	376.9	13.49	0.059
C18:1n9t	14.42 a	41.52 b	1.762	0.004
C19:1	2.930	6.296	0.229	0.225
C20:1	2.017	4.333	0.201	0.250
C22:1n9	1.226	2.617	0.043	0.465
MUFA	238.6 a	529.1 b	17.72	0.031
C18:3n3	48.21	92.12	4.075	0.519
C20:3n3	1.012	1.897	0.052	0.087
C20:5n3	45.97 a	80.90 b	3.935	0.039
C22:6n3	16.73 a	27.85 b	1.710	0.036
n-3 PUFA	111.9	202.8	9.695	0.126
C18:2n6c	110.6	208.0	9.712	0.456
C18:3n6	1.406	2.742	0.145	0.746
C20:3n6	8.008 a	14.48 b	0.442	0.042
C20:4n6	59.94	120.59	6.414	0.938
n-6 PUFA	180.0	345.8	16.62	0.600
PUFA	291.9	548.6	26.20	0.382
PUFA/SFA	0.639	0.525	0.038	0.065

^*a*^ SFA: C6:0 + C8:0 + C9:0 + C10:0 + C11:0 + C12:0 + C13:0 + C14:0 + C15:0 + C16:0 + C17:0 + C18:0 + C20:0 + C21:0 + C22:0 + C23:0 + C24:0; MUFA: C14:1 + C15:1 + C16:1 + C18:1n9c + C18:1n9t + C19:1 + C20:1 + C22:1n9; n-3 PUFA: C18:3n3 + C20:3n3 + C20:5n3 + C22:6n3; n-6 PUFA: C18:2n6c + C18:3n6 + C20:3n6 + C20:4n6; PUFA: n-3 PUFA + n-6 PUFA. a and b indicate that values within a row with different subscripts differ when the *P*-value is <0.05.

### Muscle amino acid composition

The results demonstrated how the LD was affected by the seasons, which helped discover the amino acid content of meat metabolites. Regarding the essential amino acids (EAAs), branched-chain amino acids (BCAAs), flavor amino acids (FAAs), non-essential amino acids (NEAAs), and total amino acids (TAAs), there were no appreciable variations between the two groups (Table 3). This implies that the LD of Xizang sheep has a slightly different amino acid profile depending on the season. Furthermore, we examined the variations in noncanonical amino acid concentrations in muscle between both groups and discovered that group SUM had significantly higher concentrations of glutathione oxidized, N-isovaleroylglycine, N-propionylglycine, and γ-aminobutyric-acid amino acids than group AUT (Table 4). Glutathione oxidized is one of them; it has antioxidant properties in cells and can enhance the meat quality of muscle cells by boosting their antioxidant capacity (30).

**TABLE 3:** Effects of different seasons on the concentration of standard amino acids in the LD (ng/g)^*a*^

Index	AUT	SUM	SEM	*P*-value
FAA				
Arg (arginine)	47,642.85	42,602.84	5,016.05	0.437
Glu (glutamic acid)	505,993.90	211,037.87	156,830.10	0.119
Gly (glycine)	107,081.48	106,238.16	7,919.54	0.944
Asn (asparagine)	9,355.23	8,337.57	1,205.56	0.494
Ala (alanine)	362,063.30 a	307,222.10 b	16,253.55	0.036
L-citrulline	187,050.84	226,844.26	22,966.25	0.308
Ser (serine)	16,165.23	15,299.09	1,883.21	0.742
Pro (proline)	35,517.85	25,413.68	4,068.28	0.064
L-ornithine	25,482.27	40,385.10	3,747.27	0.196
Asp (aspartic acid)	10,117.08	6,378.37	1,754.02	0.093
Tyr (tyrosine)	12,421.96	8,834.69	838.28	0.052
EAA				
Thr (threonine)	20,379.41	19,337.21	1,971.00	0.765
Phe (phenylalanine)	6,244.47	5,480.87	889.64	0.456
Met (methionine)	1,910.61	1,571.09	222.63	0.264
Lys (lysine)	10,477.48	9,050.00	1,057.55	0.320
Trp (tryptophan)	5,749.86	4,404.39	556.95	0.063
His (histidine)	28,641.32	25,899.55	2,241.20	0.571
BCAA				
Val (valine)	26,949.617	23,805.894	1,576.17	0.254
Ile (isoleucine)	13,793.431	12,692.128	1,703.97	0.589
Leu (leucine)	27,641.21	29,459.74	3,933.39	0.717
TAA	1,470,034.60	1,138,632.11	187,395.40	0.123
NEAAs	1,328,247.21	1,006,931.26	193,784.30	0.141
EAA	141,787.39	131,700.85	12,438.95	0.587
BCAA	68,384.26	65,957.76	6,881.81	0.794
FAA	1,041,491.98	683,776.07	163,679.60	0.059

^*a*^ FAA: Arg + Glu + Gly + Asn + Asn + Ala NEAA: FAA + L-citrulline + Ser + Pro + L-ornithine + Asp + Tyr BCAA: Val + Ile + Leu EAA: BCAA + Thr + Phe + Met + Lys + Trp + His TAA: EAA + NEAA. a and b indicate that values within a row with different subscripts differ when the *P*-value is <0.05.

**TABLE 4:** Effects of different seasons on the concentration of non-standard amino acids in the LD (ng/g)^*a*^

Index	AUT	SUM	SEM	*P*-value
Methionine sulfoxide	2,884.46 a	407.01 b	437.74	0.002
5-Aminovaleric acid	430.48 a	2,097.22 b	85.62	0.003
Glutathione oxidized	183,997.07 a	614,639.69 b	30,788.05	0.005
N-isovaleroylglycine	0.18 a	71.28 b	0.18	0.008
(5-L-glutamyl)-L-alanine	3,962.00 a	1,382.76 b	474.83	0.010
γ-Glutamate-cysteine	17,941.48 a	7,830.57 b	2,717.69	0.012
N-propionylglycine	9.60 a	502.73 b	9.60	0.015
γ-Aminobutyric acid	18,093.76 a	49,469.82 b	1,996.97	0.020
α-Aminoadipic acid	60,235.87 a	27,217.06 b	10,231.04	0.023
Trimethylamine-N-oxide	124.65 a	598.95 b	5.52	0.024
3-Hydroxyhippuric acid	0.00 a	282.33 b	0.00	0.031

^*a*^ a and b indicate that values within a row with different subscripts differ when the *P*-value is <0.05.

### Correlation analysis

**Fig 6:**
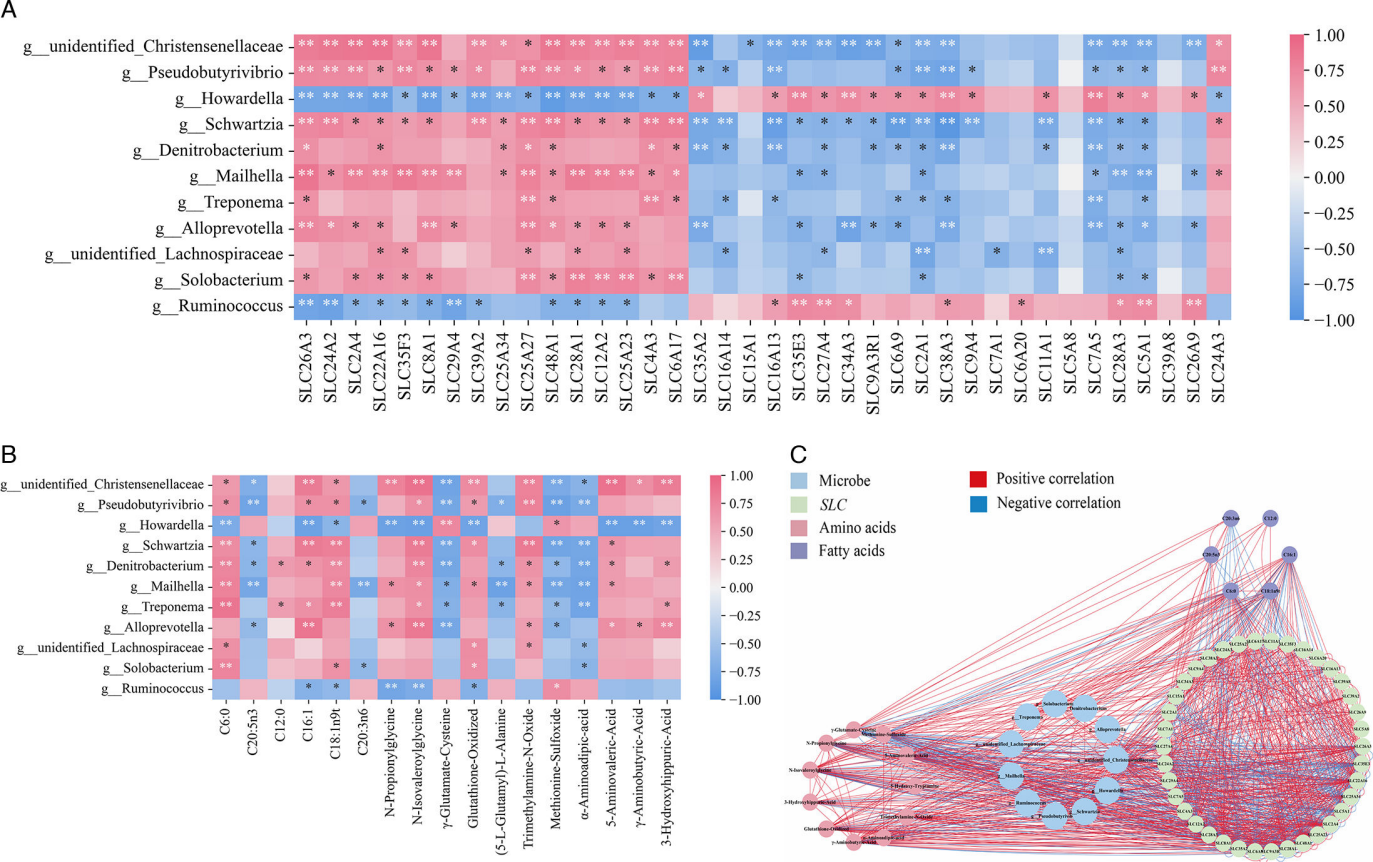
Correlation analysis of rumen microbiota with genes and muscle metabolites. ⁎ denotes significant, ⁎⁎ denotes highly significant, and ns denotes insignificant. (A) Association analysis of microbes with *SLC* genes. (B) Association analysis of microbes with muscle metabolites (amino acids and fatty acids). (C) Visual network analysis of Spearman’s correlation between rumen bacteria and rumen epithelial *SLC* genes, amino acids, and fatty acids in muscle in Xizang sheep.

We constructed heat maps and visualized network interactions of Xizang sheep rumen microbiota (microbes with significant differences at the genus level) associated with genes, muscle fatty acids, and amino acids. *Pseudobutyrivibrio* and *unidentified_Christensenellaceae* among them had significantly negative correlations with solute carrier family 2 member 1 (*SLC2A1*) and solute carrier family 38 member 3 (*SLC38A3*) (*P* < 0.01) and considerably positive correlations with *SLC26A3*, solute carrier family 24 member 2 (*SLC24A2*), solute carrier family 2 member 4 (*SLC2A4*), solute carrier family 4 member 3 (*SLC4A3*), and solute carrier family 6 member 17 (*SLC6A17*) (*P* < 0.01) (Fig. 6A). *Treponema* showed a substantial negative correlation (*P* < 0.01) with solute carrier family 7 member 5 (*SLC7A5*) and a significantly positive correlation with *SLC26A3* (*P* < 0.05) (Fig. 6A).

Regarding C6:0 and C16:1, *Howardella* had a strong negative correlation (*P* < 0.05), and it also had a negative correlation with C20:5n3 and C20:3n6 (Fig. 6B). The correlation between *unidentified_Christensenellaceae* and C16:1 was considerably positive (*P* < 0.01). Relative to C6:0, *Treponema*, *Schwartzia*, *Denitrobacterium*, and *Mailhella* showed a substantial positive correlation (*P* < 0.01) (Fig. 6B). The *Ruminococcus* and C18:1n9t and C16:1 showed a substantial negative correlation (*P* < 0.01) (Fig. 6B).

*Pseudobutyrivibrio*, *Mailhella*, and *Treponema* were shown to be strongly negatively linked with Glu in the association between amino acids and rumen microbiota (*P* < 0.05) (Fig. 6B). *Unidentified_Christensenellaceae* exhibited a substantial negative correlation (*P* < 0.01) with methionine sulfoxide and a significant positive correlation (*P* < 0.01) with trimethylamine-N-oxide, 5-aminovaleric acid, and 3-hydroxyhippuric acid (Fig. 6B). *Schwartzia* showed a substantial negative correlation with α-aminoadipic acid (*P* < 0.01) and a significant positive correlation with trimethylamine-N-oxide (*P* < 0.01) (Fig. 6B). The research mentioned above indicates a complicated relationship between genes, muscle metabolites, and the rumen bacteria (Fig. 6C).

## DISCUSSION

Consumers have recently paid special attention to the flavor of animal-derived foods. The gastrointestinal bacterial communities of ruminant animals affect muscle flavor, but the underlying mechanisms are not yet clear. Therefore, exploring the IMF deposition mechanism of the rumen-muscle axis in Xizang sheep and its connection with muscle flavor is of great significance. In this study, we found that Xizang sheep with different muscle fatty acid content have different rumen bacterial community compositions; these differential bacterial communities produce SCFAs that are absorbed by the rumen epithelium under the regulation of the *SLC* family genes; SCFAs directly reach muscle tissue through the circulatory system to regulate IMF deposition, further affecting the flavor of mutton. Thus, these results preliminarily prove that there is a direct link between rumen microbiota and mutton flavor in Xizang sheep.

Rumen bacteria possess remarkable capabilities in degrading cellulose, hemicellulose, starch, and fats. Firmicutes (31), Proteobacteria (32), Ruminococcaceae (33), and Christensenellaceae (34) play a crucial role in the degradation of complex carbohydrates, such as cellulose, hemicellulose, and starch. Acetic acid, propionic acid, butyric acid, and other SCFAs are the primary fermentation products of these substrates. The Firmicutes and Bacteroidetes are the most prevalent bacteria in ruminant animals’ rumens, holding a prominent place in the rumen’s microbial ecology (35). In particular, an increase in the abundance of Firmicutes is associated with the promotion of fat deposition in mammals. The ratio of Firmicutes to Bacteroidetes has a positive correlation with the degree of fat deposition; the higher the ratio, the more pronounced the fat deposition (35, 36). In the rumen, a higher ratio of Firmicutes to Bacteroidetes is associated with higher levels of SCFAs (37, 38). Consistent with the above results, in this study, the proportion of Firmicutes and Bacteroidota, as well as the T-SCFAs, in group SUM is greater than that in group AUT. The digestion of lipids in the rumen is essentially carried out under the action of microbes, with the majority of unsaturated fatty acids (UFAs) being hydrogenated by microbes into saturated fatty acids (39). *Christensenellaceae_R-7_group* (38) and *Lachnospiraceae* (40) can achieve fat degradation and produce fatty acids. The fat content in summer pasture is relatively high, which may lead to more active degradation of fat in the rumen fluid. Bacteria can hydrogenate to generate intermediates of UFAs, affecting the composition of UFAs in the IMF of Xizang sheep. As a key player in the biological hydrogenation process, *Butyrivibrio* can change UFAs into SFAs, which affects the UFA composition of Xizang sheep’s IMF (41). The main hydrogenating bacterium for C18:2n6 in the rumen has been identified as *Butyrivibrio fibrisolvens*, and it plays a crucial part in fat metabolism (42). Furthermore, the rumen’s biohydrogenation of fatty acids is a function of *Pseudobutyrivibrio*, *unidentified_Lachnospiraceae*, *Schwartzia*, *Denitrobacterium*, *Treponema*, and *Lachnospiraceae* (43–47). In this study, the relative abundance of *g_Pseudobutyrivibrio*, *unidentified_Lachnospiraceae*, *Schwartzia*, *Denitrobacterium*, *Treponema*, and *g_unidentified_Lachnospiraceae* in group SUM was significantly higher than in group AUT. This microbial composition is favorable for promoting the hydrogenation of UFAs in Xizang sheep. Additionally, our study revealed a complex relationship between the rumen microbiota and fatty acids. For instance, *unidentified_Christensenellaceae* showed a significant positive correlation with C16:1, whereas C6:0 was significantly positively correlated with *Treponema*. These findings suggest that these rumen microbial communities play a critical role in regulating intramuscular fat deposition in Xizang sheep.

Rumen bacteria are vital to the host’s physiological functions, and through the intermediate products they generate, their metabolic activities can have a major effect on the host’s energy metabolism (48, 49). Specifically, these bacteria’s biological fermentation produces SCFAs, which are important for promoting fat deposition. Acetic acid, propionic acid, butyric acid, and other SCFAs are important mediators in this process that control muscle metabolism (37). SCFAs have a complex role in fat deposition; they can influence angiogenesis, triglyceride synthesis, and adipocyte differentiation and are also involved in the regulation of late neonatal adipose tissue development in mammals (50, 51). Research indicated that in the rumen of yaks, the concentrations of acetic acid, butyric acid, valeric acid, and T-SCFAs are highly positively correlated with the absolute content of SFAs, UFAs, and total fat in the muscle of yaks (37). Additionally, studies have shown that infusing SCFAs into the ileum can improve lipid metabolism in growing pigs (52). Based on this finding, we can infer that during the digestion of cellulose and hemicellulose in Xizang sheep, rumen bacteria play a key role, and the SCFAs produced by their fermentation action are the main metabolic products entering the circulation of Xizang sheep. The SCFAs are primarily absorbed by the rumen wall, enter the circulatory system, and then reach the muscles to exert their effects. The absorption and metabolism of SCFAs in the rumen are complex physiological processes involving the regulation of various genes and proteins, which play a key role in maintaining the homeostasis of the rumen environment and the host’s energy metabolism (53). In this study, through comparative transcriptome sequencing of rumen epithelial tissues from two groups of grazing Xizang sheep, a total of 2,214 DEGs were found between the two groups, with 1,269 genes significantly upregulated and 945 genes significantly downregulated. Interestingly, we discovered important genes involved in the regulation of SCFA absorption in the rumen of Xizang sheep among these DEGs. There are 17 upregulated and 21 downregulated genes in the rumen epithelial *SLC* gene family, indicating distinct expression patterns. It has been discovered that *SLC16A1*, which is found in the basolateral pole of sheep rumen epithelial cells, contributes to the absorption of SCFAs (25, 54). In this study, it was found that the *SLC16A14* and *SLC16A13* genes were significantly upregulated in the rumen epithelium of group Xizang sheep, which is conducive to promoting their absorption of SCFAs. Aschenbach et al. (55) suggested that *SLC* genes encoding anion exchangers could be candidates for SCFA uptake transport proteins, including members of the *SLC4A*, *SLC21A*, *SLC22A*, and *SLC26A* series (56, 57). Nishihara et al. (58) found that genes involved in the absorption and metabolism of SCFAs respond to higher rumen SCFA concentrations, including *SLC26A3*, *SLC9C1*, *SLC26A6*, and *SLC25A21* genes. In this study, it was discovered that the *SLC4A3*, *SLC26A3*, and *SLC22A16* genes were significantly upregulated in group AUT, while the *SLC26A9* gene was significantly upregulated in group SUM. It is speculated that the expression of these genes is related to changes in the concentration of SCFAs in the rumen. Therefore, SCFAs produced by the microbial community in the rumen are absorbed by the rumen wall under the regulation of the *SLC* family genes, and the host’s direct utilization of SCFAs can achieve the synthesis of IMF in muscles, thereby affecting the flavor of sheep meat.

In muscle, the fatty acid composition is regulated by the expression of related genes. In a population of goat kids, four markers (*SCD2*, *SCD3 172*, *SCD3 181*, and *SCD3 231*) were detected in the *SCD* gene, and significant associations were found between these markers and five specific fatty acids (C8:0, C11:0, C15:1, C16:1cis-9, and CLA 10 *trans*-12*cis*), as well as two categories of fatty acids (SFA and MUFA) (59). Additionally, in Kazak male sheep, the mRNA expression levels of the *FAS* gene were negatively correlated with intramuscular IMF content (60). Research indicates that the rumen microbial community has the capability to enhance IMF deposition (61). Our study demonstrated that during summer, the concentration of SCFAs produced by the rumen microbiota in Xizang sheep was higher, and the expression levels of the *FAS* and *SCD1* genes in muscle tissue were significantly increased. Therefore, it can be inferred that the IMF fatty acid composition in Xizang sheep is closely related to the structure of the rumen microbial community.

**Fig 7:**
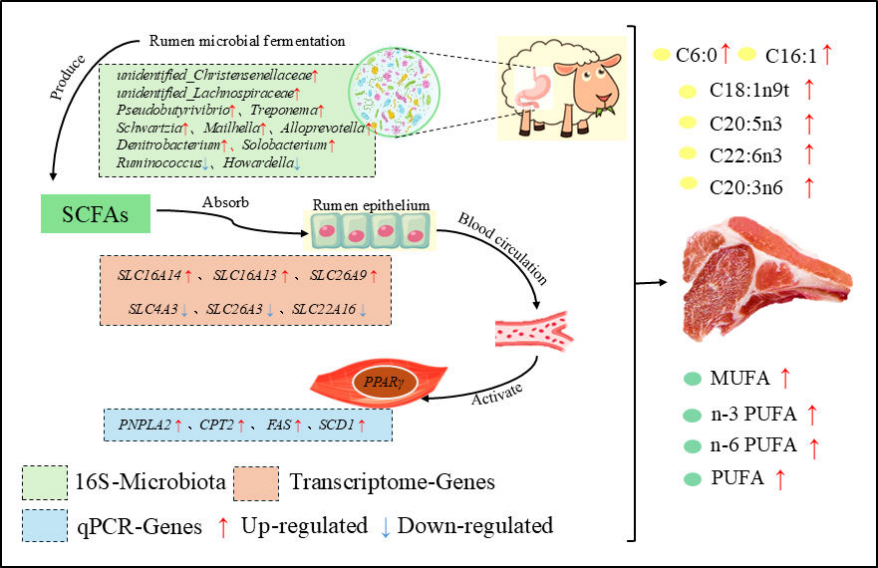
The schematic diagram of the microbial-rumen-muscle axis mediated by SCFAs regulating the fat deposition in the muscle of grazing Xizang sheep. Green squares represent the microbial communities that are significantly different at the genus level in the rumen. Orange squares represent the DEGs identified in the rumen epithelium. Blue squares represent the DEGs identified in the muscle tissue.

It is currently unclear how the rumen microbiota affects meat quality traits, despite the fact that these changes are strongly linked to gene expression. In this study, group SUM showed significantly higher expression of the *CPT2*, *PNALA2*, *FAS*, *PPARγ*, and *SCD1* genes than group AUT, while group SUM showed significantly lower expression of the *UCP2* gene. The *CPT2* gene has a regulatory role in lipid metabolism by transcriptionally activating enzymes involved in fatty acid metabolism and triglyceride breakdown, which in turn controls the expression of related enzymes (62). Rincon et al. (63) found that the expression of the *SCD* gene and the activity of its product determine the synthesis of monounsaturated fatty acids in adipocytes and the composition of phospholipids and triglycerides in cell membranes. The *SCD1* gene can regulate milk fat synthesis through the sterol regulatory element-binding protein pathway (63). Gu et al. (64) demonstrated that the specific overexpression of *PPARγ* in pig skeletal muscle can promote the formation of oxidative fibers and intramuscular fat deposition. Xiong et al. (65) found that the upregulation of *PPARγ* promoted a higher level of lipogenesis in the liver, contributing to greater body fat accumulation in the Mexican tetra (*Astyanax mexicanus*) population. Therefore, we hypothesize here that the microbe-rumen-muscle axis regulates the meat quality and flavor of grazing Xizang sheep to better investigate the relationship between rumen bacteria and genes related to lipid metabolism. Intriguingly, consistent with the hypothesis, we discovered that bacterial metabolic products, SCFAs, are absorbed under the control of the *SLC* gene family, penetrate the circulation into the muscle tissue, and influence the expression of genes linked to muscle lipid metabolism. In mammalian muscle, SCFAs have the ability to bind to free fatty acid receptors. Peroxisome proliferator-activated receptors (PPARs) are ligand-activated transcription factors that can increase the utilization of fat and glucose in mammals (66, 67). *PPARγ* can be activated by SCFAs (68, 69), and SCFAs selectively induce the expression of genes related to fatty acid uptake and β-oxidation (such as *CPT1* [70]) in an upregulated manner, subsequently increasing fatty acid synthesis in mammals (71). *PPARγ* positively regulates the expression of the *FAS* ligand gene. *SCD1* can significantly affect the fatty acid composition and synthesis rate of triglycerides through the direct regulation of *SREBP-1* and *PPARγ-1. PPARγ* agonists induce *SCD1* to weaken palmitate-induced endoplasmic reticulum stress and apoptosis (72, 73). Studies have shown that *PPARγ* regulates genes involved in the synthesis and secretion of triglycerides in goat mammary epithelial cells (such as *PNPLA2*) (74). Therefore, a hypothesis has been proposed that the deposition of IMF in Xizang sheep is regulated by the microbiome-rumen-muscle axis, meaning that SCFAs can directly reach muscle tissue through the circulatory system of Xizang sheep, activate the expression of the *PPARγ* gene through the gut-muscle axis, and under the regulation of the transcription factor *PPARγ*, the expression of lipid synthesis-related genes, such as *CPT2*, *FAS*, *PNPLA2*, and *SCD1,* is upregulated (Fig. 7).

### Conclusion

In conclusion, we discovered that the muscle of Xizang sheep grazing in the summer has a higher fatty acid content and a stronger capacity for lipid metabolism, and we put forth the hypothesis that the microbe-rumen-muscle axis regulates the fat deposition in the muscle of Xizang sheep. The rumen bacterial community compositions and rumen epithelial transcriptome expression profiles of Xizang sheep with varying muscle fatty acid contents have different compositions. Bacteria such as *Treponema*, *Pseudobutyrivibrio*, and *unidentified_Lachnospiraceae* can ferment and produce a large amount of SCFAs, which are then absorbed under the regulation of genes like *SLC4A3*, *SLC26A3*, *SLC22A1*, *SLC26A9*, *SLC16A14*, and *SLC16A13* in the rumen epithelium of Xizang sheep. SCFAs can directly reach muscle tissue through the bloodstream of Xizang sheep and activate the expression of the *PPARγ* gene through the rumen-muscle axis. Under the regulation of the transcription factor *PPARγ*, the expression of genes related to fat formation, such as *CPT2*, *FAS*, *PNPLA2,* and *SCD1,* is upregulated, thereby promoting fatty acid synthesis and increasing the muscle fat content of Xizang sheep. This study provides new insights into the fat deposition of grazing sheep in high-altitude areas and offers relevant references for their production management.

## Data Availability

All data in this study are available upon request from the corresponding author. The sequencing data for Xizang sheep have been deposited in the NCBI Sequence Read Archive (SRA) under the project accessions PRJNA1142199 and PRJNA1142721.

## SUPPLEMENTAL MATERIAL

Supplemental material msystems.01557-24-s0001.docx Tables S1 to S4 and Fig. S1 legend.

Fig. S1A msystems.01557-24-s0002.tif Expression and functional analysis of *SLC* gene in rumen epithelium: heat maps.

Fig. S1B msystems.01557-24-s0003.tiff Expression and functional analysis of *SLC* gene in rumen epithelium: volcano plots.

Fig. S1C msystems.01557-24-s0004.tiff Expression and functional analysis of *SLC* gene in rumen epithelium: KEGG pathway map.

Fig. S1D msystems.01557-24-s0005.tiff Expression and functional analysis of *SLC* gene in rumen epithelium: correlation analysis.

ASM does not own the copyrights to Supplemental Material that may be linked to, or accessed through, an article. The authors have granted ASM a non-exclusive, world-wide license to publish the Supplemental Material files. Please contact the corresponding author directly for reuse.
