# Supplementary material for: Rumen microbiota regulates IMF deposition in Xizang sheep by activating the PPARγ transcription factor: a rumen-muscle axis perspective
Source: mSystems. 2025 Mar 28;10(4):e01557-24. doi: 10.1128/msystems.01557-24 (PMC12013263; doi:10.1128/msystems.01557-24)
Supplement: Supplemental material — Tables S1 to S4 and Fig. S1 legend. [file msystems.01557-24-s0001.docx]

**Supplementary Table 1**.Primers used for Quantitative Real-Time PCR

| **Genes** | **Sequence(5'→3'）** | **Product size/bp** |
| --- | --- | --- |
| *GAPDH* | F: CCTGCCAAGTATGATGAGAT | 117 |
|  | R: AGTGTCGCTGTTGAAGTC |  |
| *MYH1* | F: AAGCAGAAGTTGAGAGTGAA | 217 |
|  | R: TTGGAGAGGTTGACATTGG |  |
| *MYH2* | F: TCGCATCCATTGACATTGA | 186 |
|  | R: TCCTCTCCACCATCCTCT |  |
| *MYH4* | F: CATCTCTGACAACGCCTAT | 161 |
|  | R: TTGGTTCCTCCTTCTTCTTC |  |
| *MYH7* | F: CGTGCTCTACAACCTCAA | 151 |
|  | R: TGCCTCACTCCTCTTCTT |  |
| *CPT2* | F: GAGCCAGAAGTGTTCCATT | 116 |
|  | R: TATGCGTTGACCAAGTAGG |  |
| *FAS* | F: ATGGCTGGTATCAACTCTG | 202 |
|  | R: GATGGCTCTTGTCTGTGTA |  |
| *PNALA2* | F: CTCGGCGTCTACCATATC | 223 |
|  | R: TCACCATGTTGAAGGAAGG |  |
| *PPARγ* | F: ATCAAGTTCAAGCACATCAG | 154 |
|  | R: CATTCAAGTCAAGGTTCACA |  |
| *SCD1* | F: AATTCCACAGGTCATCAGAT | 310 |
|  | R: GTAAGGCATCCAGATAAGTTG |  |
| *UCP2* | F: CGGACACATAGTATGACCAT | 195 |
|  | R: CAGCAACAAGACGAGACA |  |
| *ABCA13* | F: CTGGAAGAGTATATGAGGATGA | 170 |
|  | R: AGGAGGAAGAGGAAGATGAT |  |
| *SLC26A3* | F: AACCTCCACAGCCTCATT | 134 |
|  | R: TCATCATCAGTTCCAGCAAT |  |
| *GKN1* | F: CATCTTCCTGACTCCTACTC | 174 |
|  | R: AAGCCACTTCCGTAATCC |  |
| *IL6* | F: TGCTGGTCTTCTGGAGTAT | 154 |
|  | R: TGTGGCTGGAGTGGTTAT |  |
| *IL17C* | F: CCCACCTTGAAAGACAGTT | 118 |
|  | R: CAGCCATCAGAACACAGT |  |
| *ACP7* | F: AGAGGAAGAGGAAGAGTAGG | 267 |
|  | R: ACAACACAATGGACGAAGT |  |
| *SLC26A9* | F: CTCATCTTCGCTCTCATCA | 112 |
|  | R: CCACTACCACAATCATCTCT |  |
| *MX2* | F: TTCAGGAGCAGAGCGATA | 267 |
|  | R: AGGTGGTTCAGTTCAGTTC |  |

**Supplementary Table 2**: Comparison of differences in SCFAs in the rumen of Xizang sheep in different seasons.

| **Index** | **Q** | **X** | **SEM** | ***P*-value** |
| --- | --- | --- | --- | --- |
| Acetic acid（mmol/L） | 117.17 a | 191.01 b | 0.91 | 0.01 |
| Propanoic acid（mmol/L） | 46.43 | 40.48 | 0.40 | 0.19 |
| Butyric acid（mmol/L） | 15.85 | 18.71 | 0.30 | 0.47 |
| Isobutyric acid（mmol/L） | 0.79 | 1.5 | 0.03 | 0.36 |
| Valeric acid（mmol/L） | 1.27 | 5.69 | 2.26 | 0.19 |
| Isovaleric acid（mmol/L） | 0.31 | 4.56 | 0.003 | 0.080 |
| T-SCFAs | 181.82 | 261.95 | 18.82 | 0.71 |

T-SCFAs = Acetic acid + Propanoic acid + Butyric acid + Isobutyric acid + Valeric acid + Isovaleric acid.

a−b means within a row with different subscripts differ when p-value < 0.05.

**Supplementary Table 3**.Effects of different seasons on the rumen microbiota at the phylum level (%).

| **Phylum** | **X** | **Q** | **SEM** | ***P*-value** |
| --- | --- | --- | --- | --- |
| unidentified_Archaea | 0.0078 | 0.0014 | 0.0053 | 0.2582 |
| unidentified_Bacteria | 0.0049 | 0.0048 | 0.0014 | 0.9574 |
| Gracilibacteria | 0.0036 | 0.0077 | 0.0007 | 0.1780 |
| Actinobacteriota | 0.0079 | 0.0056 | 0.0035 | 0.5829 |
| Euryarchaeota | 0.0089 | 0.0144 | 0.0020 | 0.4500 |
| Proteobacteria | 0.0126 | 0.0133 | 0.0089 | 0.9512 |
| Spirochaetota | 0.0184 | 0.0126 | 0.0038 | 0.3078 |
| Fibrobacterota | 0.0200 | 0.0111 | 0.0043 | 0.1026 |
| Firmicutes | 0.2799 | 0.2510 | 0.0105 | 0.1772 |
| Bacteroidota | 0.6238 | 0.6666 | 0.0188 | 0.1964 |
| Others | 0.0123 | 0.0116 | 0.0021 | 0.8236 |

**Supplementary Table 4**.Effects of different seasons on the rumen microbiota at the genus level (%).

| **Genus** | **X** | **Q** | **SEM** | ***P*-value** |
| --- | --- | --- | --- | --- |
| *Ruminococcus* | 0.0053 a | 0.0091 b | 0.0006 | 0.0281 |
| *Succiniclasticum* | 0.0099 | 0.0063 | 0.0025 | 0.2621 |
| *Saccharofermentans* | 0.0100 | 0.0090 | 0.0012 | 0.6221 |
| *Treponema* | 0.0150 a | 0.0054 b | 0.0031 | 0.0245 |
| *Methanobrevibacter* | 0.0078 | 0.0132 | 0.0018 | 0.4212 |
| *Papillibacter* | 0.0106 | 0.0114 | 0.0022 | 0.7642 |
| *Selenomonas* | 0.0195 a | 0.0074 b | 0.0052 | 0.0496 |
| *Fibrobacter* | 0.0200 | 0.0107 | 0.0043 | 0.0867 |
| *unidentified_Bacteroidales* | 0.0127 | 0.0208 | 0.0023 | 0.2264 |
| *Prevotella* | 0.1758 a | 0.1844 b | 0.0006 | 0.0281 |
| Others | 0.7134 | 0.7224 | 0.0189 | 0.8165 |

a−b means within a row with different subscripts differ when *p*-value < 0.05.

**Supplementary Figure Legends**

**Supplementary Fig. 1**. Expression and functional analysis of *SLC* gene in rumen epithelium.(A) Heat maps were drawn to visualize the expression of rumen epithelial *SLC* genes in each group. (B) volcanic map. (C) KEGG pathway map. (D) Correlation analysis was used to explore the relationship between *SLC* genes.
