# Supplementary figures and images for: Rumen microbiota regulates IMF deposition in Xizang sheep by activating the PPARγ transcription factor: a rumen-muscle axis perspective
Source: mSystems. 2025 Mar 28;10(4):e01557-24. doi: 10.1128/msystems.01557-24 (PMC12013263; doi:10.1128/msystems.01557-24)

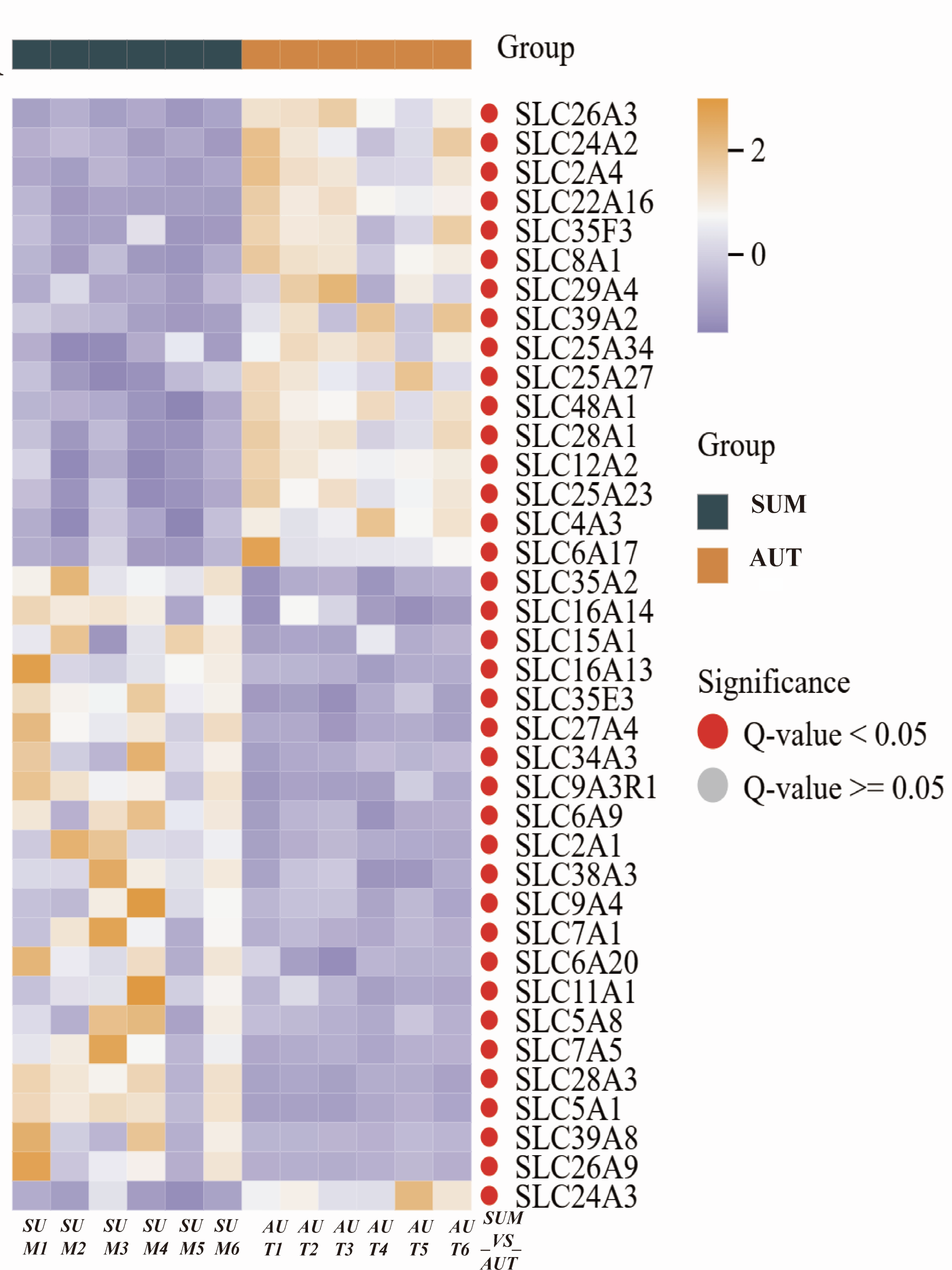

Supplement: Fig. S1A — Expression and functional analysis of SLC gene in rumen epithelium: heat maps. [file msystems.01557-24-s0002.tif]

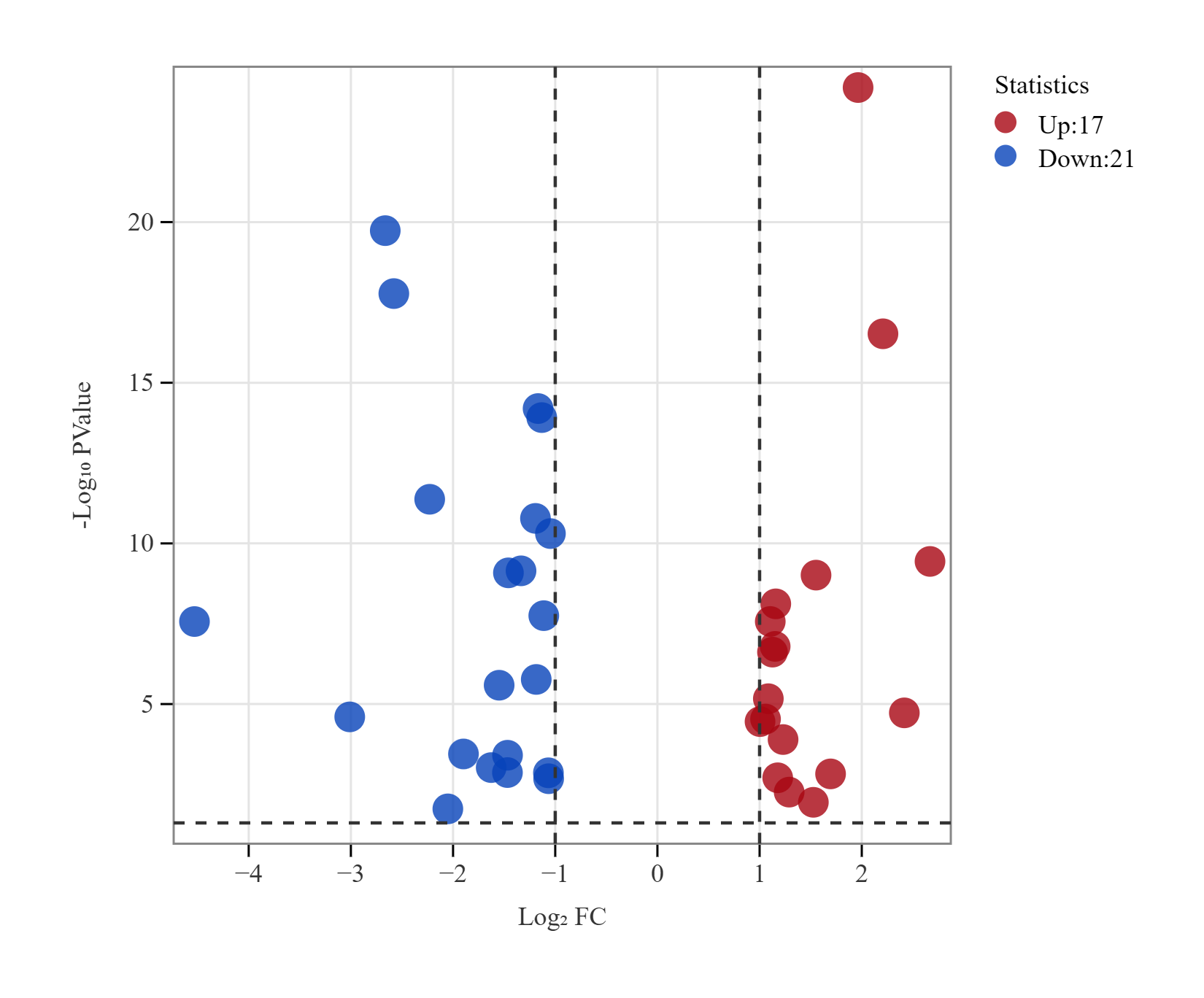

Supplement: Fig. S1B — Expression and functional analysis of SLC gene in rumen epithelium: volcano plots. [file msystems.01557-24-s0003.tiff]

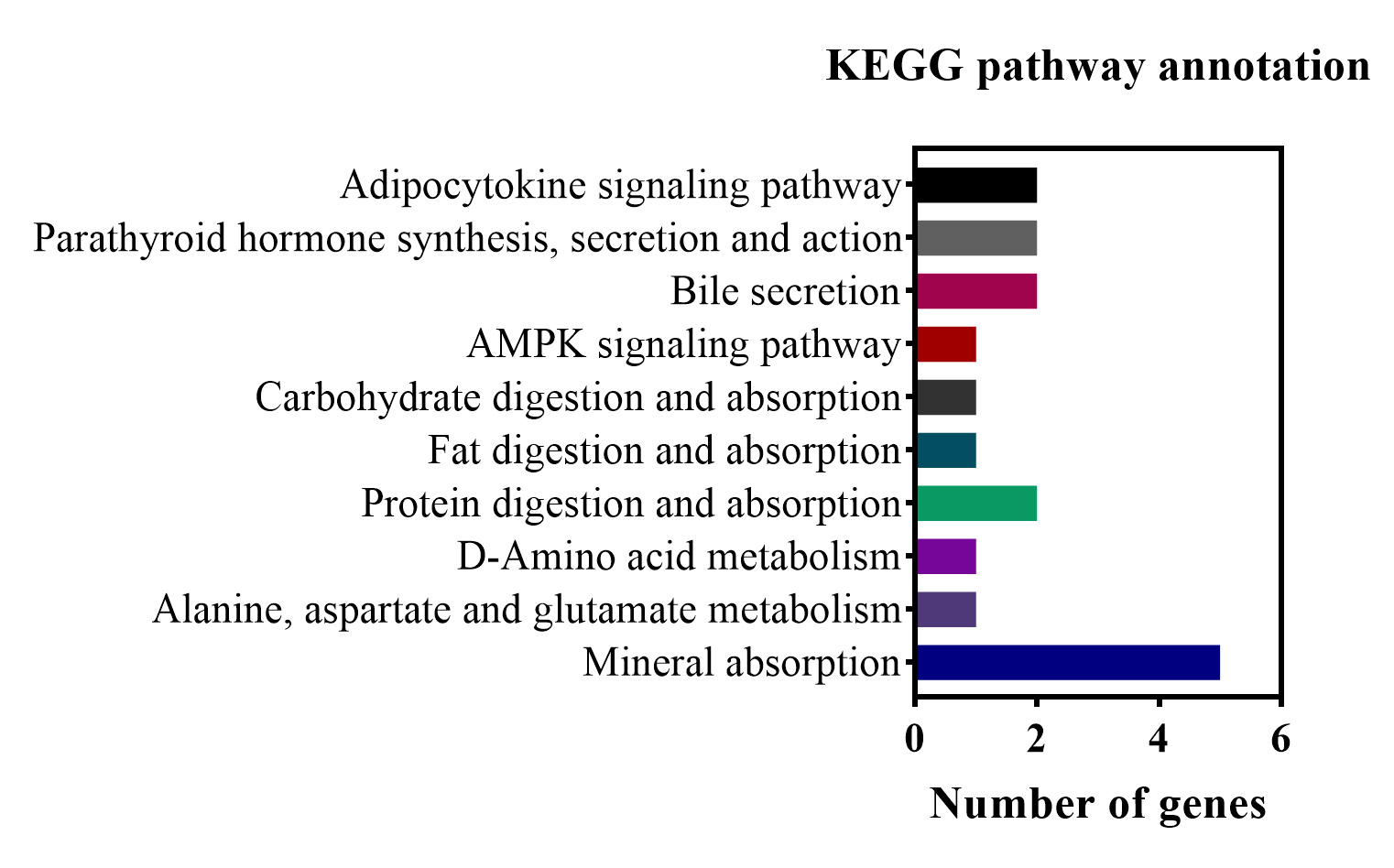

Supplement: Fig. S1C — Expression and functional analysis of SLC gene in rumen epithelium: KEGG pathway map. [file msystems.01557-24-s0004.tiff]

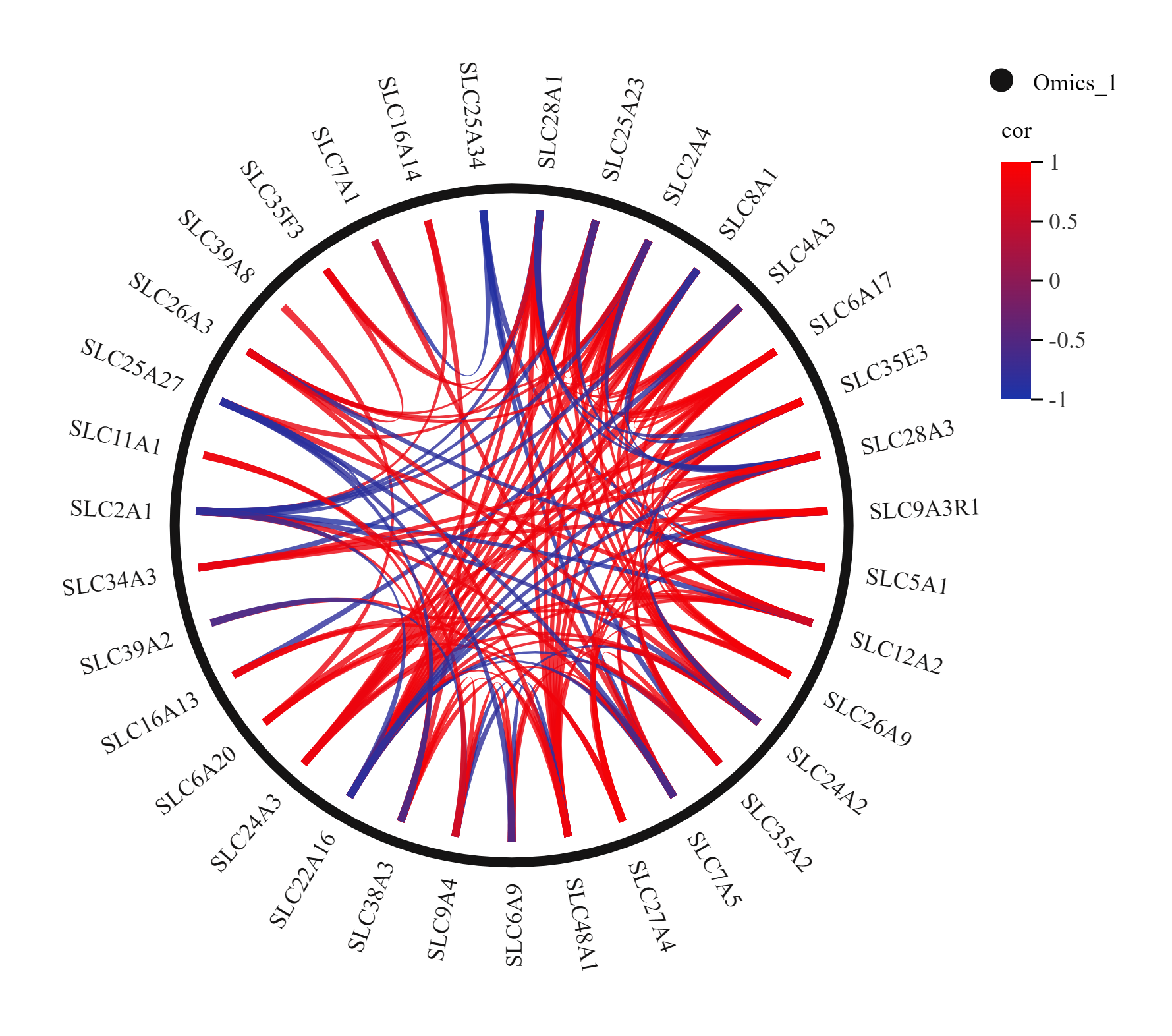

Supplement: Fig. S1D — Expression and functional analysis of SLC gene in rumen epithelium: correlation analysis. [file msystems.01557-24-s0005.tiff]
